# Major response with sorafenib in advanced renal cell carcinoma after 14 years of follow-up

**DOI:** 10.1186/1477-7819-11-243

**Published:** 2013-09-27

**Authors:** Mathilde Guerin, Naji Salem, Jochen Walz, Slimane Dermeche, Gwenaelle Gravis

**Affiliations:** 1 Department of Medical Oncology, Institut Paoli-Calmettes, Marseille, France; 2 Department of Radiotherapy, Institut Paoli-Calmettes, Marseille, France; 3 Department of Oncologic Urology, Institut Paoli-Calmettes, Marseille, France

**Keywords:** Kidney cancer, Targeted therapy, Prolonged response

## Abstract

Tyrosine kinase inhibitors have dramatically improved the prognosis of metastatic renal cell carcinoma (RCC). However, it remains unknown whether treatment should be continued until progression or discontinued in patients with good response. We present the history of a woman diagnosed with RCC in 1997, who started sorafenib in 2004, two years after the occurrence of lung and mediastinal metastases. Over the following 8 years, the sorafenib dose was reduced at least 3 times due to toxicity and the treatment was discontinued twice upon the patient’s decision, from May 2005 to March 2009, then from January 2011 to August 2011. The last evaluation in January 2013 showed stable disease. This case illustrates the feasibility of treatment discontinuation without negative impact on survival, as previously shown by some authors.

## Background

Renal cell carcinoma (RCC) represents only 3% of all cancers but the incidence is increasing worldwide. The poor prognosis of the disease, due to chemo-insensitivity, was dramatically improved by tyrosine kinase inhibitors (TKIs). First-line treatment currently consists of sunitinib or interferon-alpha plus bevacizumab [1,2], and sorafenib is considered as a second-line option [3,4].

TKIs have demonstrated significant benefits, with high rates of partial response (PR) but median survival at 5 years is less than 10%. Moreover, there is no consensus on the strategy to be chosen in responders with regards to treatment continuation or discontinuation. We report here a major and prolonged clinical response following sorafenib treatment in a patient with pulmonary, mediastinal and brain metastases of clear cell RCC, still alive 14 years after diagnosis.

## Case presentation

In April 1997, a 31-year-old woman was diagnosed with a clear cell RCC, Fuhrman nuclear grade 1. After radical right nephrectomy, the tumor was staged pT3N0M0. In 2002, lung metastases were discovered and histologically confirmed. The patient received a combination of interferon and interleukin-2 for 2 years. She had stable disease after 6 months of this treatment. In February 2004, she relapsed with mediastinal nodes. The patient was in the intermediate risk group because of hypercalcemia. Other biological parameters considered as prognostic factors in the MSKCC [5,6] and the Heng [7] risk classifications were normal (serum hemoglobin, serum lactate deshydrogenase, neutrophils and platelets); Karnosfsky performance status and time from diagnosis to treatment were also in the low-risk category.

She was included in a phase III trial [3] and treated with sorafenib 400 mg *bid*. After three weeks, the dose was reduced to 200 mg *bid* due to cutaneous toxicity, then resumed at 400 mg *bid*. After 3 months, she had stable disease (-23% according to RECIST criteria) and after 1 year, in February 2005, the patient was in partial response (-33% according to RECIST criteria). This treatment was pursued until May 2005. At this time, the patient presented with two symptomatic brain metastases, treated by surgery and whole-brain radiotherapy. Histology confirmed the RCC origin. Other lesions were stable and the patient decided to stop systemic therapy for personal reasons.

**Figure 1:**
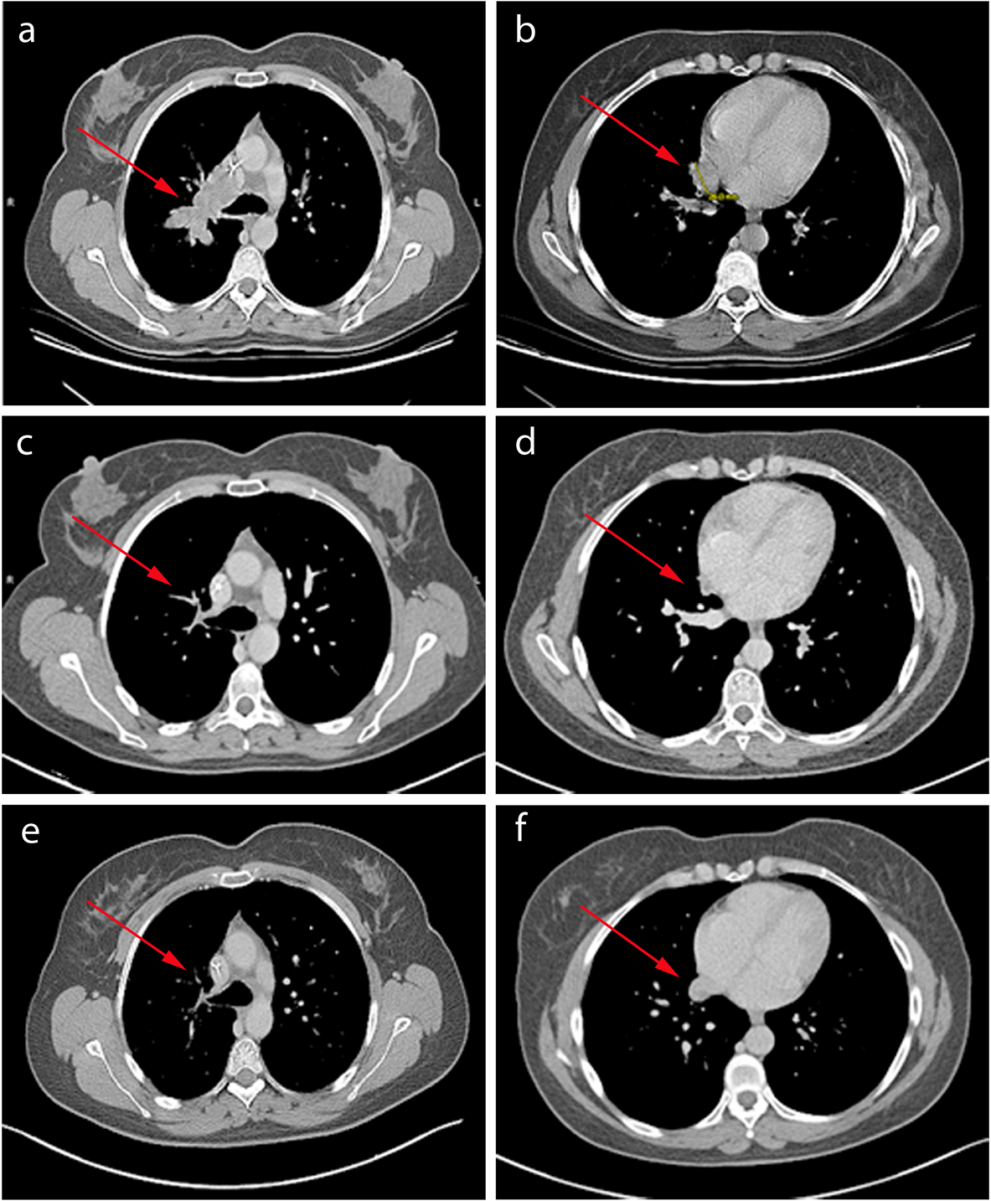
**Tumor shrinkage assessed by computed tomography. (a** and **b)** Mediastinal and chest progression without treatment in March 2009; **(c** and **d)** tumor shrinkage of 90% according to RECIST criteria in January 2011 after 10 months of Sorafenib; **(e** and **f)** stable disease in January 2013.

In March 2009, mediastinal and chest progression was observed (+60% according to RECIST criteria) (Figure 1a and b). Treatment with Sorafenib was restarted 400 mg *bid*, but a new dose reduction at 200 mg *bid* was necessary due to grade 2 to 3 hand-foot toxicity; moreover, alopecia required frequent interruptions of treatment.

After six months, tumor shrinkage of 50% was observed. The patient discontinued the treatment again in January 2011 (Figure 1c and d). At this time, she exhibited a major response (-90% according to RECIST criteria as compared to March 2009). Three pulmonary nodes appeared in August 2011 and the patient resumed sorafenib 200 mg *bid*, with stable disease at last evaluation in January 2013 (Figure 1e and f).

## Discussion

The approval of sorafenib and sunitinib opened a new era in the treatment of advanced RCC. However, complete remissions are rare and only reported in retrospective studies [8,9], mostly with sunitinib, sometimes associated with local treatment. Until recently, recommendations were to continue targeted therapies as long as the disease does not progress. However, all studies and case reports show a significant toxicity, which negatively impacts the patient’s quality of life. Moreover, since TKIs improve the outcome of cancer patients, increase survival, and are thus prescribed for several months or years, long-term administration could become a public health issue due to the high cost of treatment. Thus, one of the main questions regarding treatment with TKIs is whether the drug should be stopped in the case of good response or continued until progression.

The advantages of treatment discontinuation include better tolerance and patient’s convenience, reduced costs and possibly, the lack of development of resistant clones. However, this strategy should not jeopardize the global outcome of treatment. In the case presented above, intermittent treatment was a relevant option, as it did not compromise prolonged survival.

Some publications also advocate discontinuation: Sadeghi *et al*. published a retrospective series of 40 patients with metastatic RCC [10] who had RECIST-defined stable disease or better on therapy, and who stopped treatment for reasons excluding progression (toxicity in most cases). The overall median progression-free survival (PFS) off therapy was 10 months (1.4 to 27.2).

Other recent retrospective studies [8,11] demonstrated that TKIs could be stopped in patients who had complete response (CR). In the study published by Albiges et al. [8], 64 patients who had CR with sunitinib were identified. Among those who achieved CR with sunitinib alone (n = 36), 28 stopped treatment and 61% of them were still in CR after median follow up of 255 days. In 24 patients who experienced disease relapse, 11 were rechallenged with the same TKI, among which 7 achieved PR and one patient had stable disease. This study did not reveal any factor that could help identify patients less likely to relapse after treatment discontinuation.

In the study published by Demiselle *et al*. [11], five patients with CR and treatment discontinuation were identified. After one year, two of them were still in CR and three patients had relapsed at 3, 12 and 15 months. In a study published by Zama *et al*. [12], 23 mRCC patients were rechallenged with sunitinib after disease progression; 22% achieved PR with median PFS of 7.2 months. The PFS was significantly longer in patients who were rechallenged after more than 6 months. Overall, these results suggest that PFS can be prolonged after treatment discontinuation in responders, while resistance to TKI does not develop during treatment-free intervals.

The possible benefit of treatment discontinuation may be based on the hypothesis that residual tumor cells remain sensitive to TKIs whereas continuous treatment could result in the development of resistant cell populations. *In vitro* and *in vivo* studies suggest that resistance to TKIs is reversible, since it may be the result of changes occurring in the tumor and/or its microenvironment rather than permanent genetic changes [13].

These findings must be confirmed in prospective studies. A phase II trial, designed to investigate intermittent sunitinib treatment, was presented at the ASCO meeting in June 2013 [14]. In this study, patients who achieved tumor regression of 10% or more after four cycles of sunitinib, discontinued treatment until progression. Treatment was maintained for other patients. Intermittent sunitinib was associated with less toxicity in responders, and clinical efficacy did not appear compromised. As in this study, our patient had more than 10% tumor regression after six months of sorafenib, but this TKI was poorly tolerated and negatively impacted her daily life. Intermittent therapy brought her significant benefits with regard to quality of life, without compromising the clinical efficacy of sorafenib, with 14 years of major response.

## Conclusion

In conclusion, TKI treatment discontinuation or new modalities of administration need to be explored. At present, the prognosis of RCC is determined by Furhman nuclear grade, MSKCC classification and more recently, the Heng classification. But these clinical and biological markers cannot predict which patients are more likely to benefit from intermittent therapy. We need to improve our knowledge in prognostic factors of therapeutic efficiency and biological predictive markers, which could help identify patients at risk of relapse.

## Consent

Written informed consent was obtained from the patient for the publication of this report and any accompanying images.

## Abbreviations

PFS: Progression-free survival; PR: Partial response; CR: Complete response; RCC: Renal cell carcinoma; TKI: Tyrosine kinase inhibitor.

## Competing interests

The authors declared that they have no competing interests.

## Authors’ contributions

GM Conception, assembly of data analysis, searched the database, selected the articles and drafted the manuscript; GG conception, provision of patient, supervised the methodology, selection of articles and the writing of the manuscript, and is the corresponding author; DS and GG performed clinical treatment; SN performed radiotherapy; WJ performed surgery, supervised the writing of the manuscript. All authors read and approved the final manuscript.

## References

[B1] Escudier B Bellmunt J Negrier S Bajetta E Melichar B Bracarda S Ravaud A Golding S Jethwa S Sneller V Phase III trial of bevacizumab plus interferon alfa-2a in patients with metastatic renal cell carcinoma (AVOREN): final analysis of overall survival J Clin Oncol 2010 28 2144 2150 10.1200/JCO.2009.26.7849 20368553

[B2] Escudier B Pluzanska A Koralewski P Ravaud A Bracarda S Szczylik C Chevreau C Filipek M Melichar B Bajetta E Gorbunova V Bay JO Bodrogi I Jagiello-Gruszfeld A Moore N Bevacizumab plus interferon alfa-2a for treatment of metastatic renal cell carcinoma: a randomised, double-blind phase III trial Lancet 2007 370 2103 2111 10.1016/S0140-6736(07)61904-7 18156031

[B3] Escudier B Eisen T Stadler WM Szczylik C Oudard S Siebels M Negrier S Chevreau C Solska E Desai AA Rolland F Demkow T Hutson TE Gore M Freeman S Schwartz B Shan M Simantov R Bukowski RM Sorafenib in advanced clear-cell renal-cell carcinoma N Engl J Med 2007 356 125 134 10.1056/NEJMoa060655 17215530

[B4] Ljungberg B Cowan NC Hanbury DC Hora M Kuczyk MA Merseburger AS Patard JJ Mulders PF Sinescu IC EAU guidelines on renal cell carcinoma: the 2010 update Eur Urol 2010 58 398 406 10.1016/j.eururo.2010.06.032 20633979

[B5] Motzer RJ Hutson TE Tomczak P Michaelson MD Bukowski RM Oudard S Negrier S Szczylik C Pili R Bjarnason GA Garcia-del-Muro X Sosman JA Solska E Wilding G Thompson JA Kim ST Chen I Huang X Figlin RA Overall survival and updated results for sunitinib compared with interferon alfa in patients with metastatic renal cell carcinoma J Clin Oncol 2009 27 3584 3590 10.1200/JCO.2008.20.1293 19487381 PMC3646307

[B6] Medical Research Council Renal Cancer Collaborators Interferon-alpha and survival in metastatic renal carcinoma: early results of a randomised controlled trial Lancet 1999 353 14 17 10023944

[B7] Heng DY Xie W Regan MM Warren MA Golshayan AR Sahi C Eigl BJ Ruether JD Cheng T North S Venner P Knox JJ Chi KN Kollmannsberger C McDermott DF Oh WK Atkins MB Bukowski RM Rini BI Choueiri TK Prognostic factors for overall survival in patients with metastatic renal cell carcinoma treated with vascular endothelial growth factor-targeted agents: results from a large, multicenter study J Clin Oncol 2009 27 5794 5799 10.1200/JCO.2008.21.4809 19826129

[B8] Albiges L Oudard S Negrier S Caty A Gravis G Joly F Duclos B Geoffrois L Rolland F Guillot A Laguerre B Legouffe E Kohser F Dietrich PY Theodore CA Escudier B Complete remission with tyrosine kinase inhibitors in renal cell carcinoma J Clin Oncol 2012 30 482 487 10.1200/JCO.2011.37.2516 22231040

[B9] Johannsen M Florcken A Bex A Roigas J Cosentino M Ficarra V Kloeters C Rief M Rogalla P Miller K Grunwald V Can tyrosine kinase inhibitors be discontinued in patients with metastatic renal cell carcinoma and a complete response to treatment? A multicentre, retrospective analysis Eur Urol 2009 55 1430 1438 10.1016/j.eururo.2008.10.021 18950936

[B10] Sadeghi S Albiges L Wood LS Black SL Gilligan TD Dreicer R Garcia JA Escudier BJ Rini BI Cessation of vascular endothelial growth factor-targeted therapy in patients with metastatic renal cell carcinoma: feasibility and clinical outcome Cancer 2012 118 3277 3282 10.1002/cncr.26666 22139966

[B11] Demiselle J Lheureux S Clarisse B Sevin E Joly F Metastatic renal cancer: evolution of five complete response cases after the antiangiogenic discontinuation Bull Cancer 2011 98 626 632 10.1684/bdc.2011.1368 21659064

[B12] Zama IN Hutson TE Elson P Cleary JM Choueiri TK Heng DY Ramaiya N Michaelson MD Garcia JA Knox JJ Escudier B Rini BI Sunitinib rechallenge in metastatic renal cell carcinoma patients Cancer 2010 116 5400 5406 10.1002/cncr.25583 21105118

[B13] Zhang L Bhasin M Schor-Bardach R Wang X Collins MP Panka D Putheti P Signoretti S Alsop DC Libermann T Atkins MB Mier JW Goldberg SN Bhatt RS Resistance of renal cell carcinoma to sorafenib is mediated by potentially reversible gene expression PLoS One 2011 6 e19144 10.1371/journal.pone.0019144 21559452 PMC3084751

[B14] Rini BI Wood LS Elson P Zhu H Chittoria N Mittal K Dreicer R Gilligan TD Shah SN Garcia JA A phase II study of intermittent sunitinib (S) in previously untreated patients (pts) with metastatic renal cell carcinoma (mRCC) ASCO Meeting Abstracts 2013 31 4515

